# Community Structure, Biodiversity and Spatiotemporal Distribution of the Black Flies (Diptera: Simuliidae) Using Malaise Traps on the Highest Mountain in Thailand

**DOI:** 10.3390/insects12060504

**Published:** 2021-05-31

**Authors:** Wichai Srisuka, Chayanit Sulin, Kittipat Aupalee, Thapanat Phankaen, Kritsana Taai, Sorawat Thongsahuan, Atiporn Saeung, Hiroyuki Takaoka

**Affiliations:** 1 Entomology Section, Queen Sirikit Botanic Garden, Chiang Mai 50180, Thailand; wsrisuka@gmail.com (W.S.); chayanitsulin@gmail.com (C.S.); 2 Center of Insect Vector Study, Department of Parasitology, Faculty of Medicine, Chiang Mai University, Chiang Mai 50200, Thailand; kittipat.aupalee@gmail.com; 3 Vector Borne Disease Control Center 1.3, Chiang Rai 57000, Thailand; tptern@hotmail.com; 4 Faculty of Veterinary Medicine, Western University, Kanchanaburi 71170, Thailand; kritsana_taai@hotmail.com; 5 Faculty of Veterinary Science, Prince of Songkla University, Songkhla 90110, Thailand; sorawat.t@psu.ac.th; 6 Higher Institution Centre of Excellence (HICoE), Tropical Infectious Diseases Research and Education Centre (TIDREC), Universiti Malaya, Kuala Lumpur 50603, Malaysia

**Keywords:** black flies, Simuliidae, *Simulium*, biodiversity, malaise trap, tropical forests

## Abstract

**Simple Summary:**

Black flies, also known as buffalo gnats, are major pests to humans and animals. Females of some black fly species serve as vectors for transmitting several pathogens (i.e., filarial nematodes, blood protozoa, viruses, and bacteria) to humans and animals via their bites. In Thailand, some human-biting species are considered as natural vectors of zoonotic onchocerciasis. This study was the first to contribute baseline data on the community structure, biodiversity and spatial and temporal distribution of adult black flies in tropical forests of the highest mountain in northern Thailand, Doi Inthanon National Park, by using malaise traps. Adult black flies were captured monthly at low to high elevation sites, using malaise traps across three seasons during a one-year period. A total of 44 species were identified among 9406 specimens. It was found that species richness was greatest at the mid elevation. Black fly populations peaked in the rainy season at all elevation sites. The findings of this study showed that varied elevations and seasons are important factors that influence the distribution and abundance of black flies in this region.

**Abstract:**

Black flies form a group of small blood-sucking insects of medical and veterinary importance. This study aimed to investigate the community structure, biodiversity and spatial and temporal distribution of adult black flies in tropical rain forests, by using malaise traps in Doi Inthanon National Park, northern Thailand. Malaise traps were placed along six elevational gradients (400 m to 2500 m, above sea level) at Doi Inthanon National Park, Chiang Mai province, from December 2013 to November 2014. A total of 9406 adult female black flies belonging to five subgenera—*Daviesellum* (2%), *Gomphostilbia* (23%), *Montisimulium* (11%), *Nevermannia* (16%) and *Simulium* (48%)—were collected. Among 44 taxa found, *S*. *tenebrosum* complex had the highest relative abundance (11.1%), followed by the *S. asakoae* species-group (9.6%), the *S. striatum* species-group (7.7%), *S. inthanonense* (6.6%), *S. doipuiense* complex (6.4%), *S. chomthongense* complex (5.3%), *S. chumpornense* (5.1%) and *S. nigrogilvum* (4.1%). Two human-biting species—*S. nigrogilvum* and species in the *S. asakoae* species-group—were found in all of the collection sites with 100% species occurrence. Species richness was highest at mid elevation (1400 m), which is represented by 19 black fly species. The peak and lowest seasonal abundance was observed in the rainy and hot season, respectively. Seasonal species richness was highest in the cold season, except for that from elevation sites at 700 m, 1700 m and 2500 m. This study revealed that the malaise trap is effective in providing important data for further monitoring of the effects of environmental changes and conservation planning on the biodiversity of black flies in Doi Inthanon National Park.

## 1. Introduction

Black flies (Diptera: Simuliidae) are among the most important groups of blood-sucking insects [1]. The bite of adult females can cause serious medical problems to humans and other vertebrates [2]. The larvae and pupae are at the aquatic stage, live in running waterways and play a role in filter-feeding and servicing the ecology [3,4,5,6]. Furthermore, some hill tribes in northern Thailand consume the larvae of *S. rudnicki* (known as Kokob salad) on special occasions [7]. At present, 2331 living black fly species have been recorded worldwide [8]. The black fly fauna has been studied in some Oriental countries, such as Indonesia (124 species), Peninsular Malaysia (63 species), Myanmar (28 species), the Philippines (84 species) and Vietnam (70 species) [8,9]. Currently, at least 139 black fly species have been reported in Thailand [10,11,12,13]. Among these, seven species have been incriminated as human-biting species—*S. asakaoe*, *S. chamlongi*, *S. doipuiense* complex, *S. nigrogilvum*, *S. nodosum*, *S. tenebrosum* complex and *S. umphangense* [2,14]. *S*. *asakoae, S. monglaense* and *S. myanmarense*, all in the *S. asakoae* species-group of the subgenus *Gomphostilbia* Enderlein, are natural vectors of unnamed filarial larvae, and *S. nodosum* and *S. nigrogilvum*, both of the subgenus *Simulium* Latreille, are natural vectors of filarial larvae in the genus *Onchocerca* [15,16,17,18,19,20].

Doi Inthanon National Park is part of the cold Himalayan mountain range that extends from India through Nepal to Thailand. Doi Inthanon is the highest mountain in Thailand at 2565 meters above sea level. It serves as a biodiversity hotspot for many animals and plants, due to its suitable microhabitat [21]. The first study of black flies (*S. nigrogilvum*) in this park was conducted by Summers (1911) and then by Edwards (1928). Subsequently, Takaoka and Suzuki (1984) reported 19 black fly species and were the first to construct a standard key to identify them in Thailand [22]. Black fly larvae have been surveyed from several localities in this park since 1999, from which 17 species were discovered [23]. The seasonal abundance and daily activity of 23 black fly species attracted to humans were reported by Choochote et al. [2]. In 2007, the larvae and pupae of 40 black fly species collected from several permanent and seasonal streams in this Park were reported in the Darwin Initiative Project [24]. Between 1911 and 2019, 56 black fly species were recorded from this area, which accounted for 32% of the total number of black flies found in Thailand [10,12,13,25]. 

The tent-like passive intercept traps, malaise traps, have been used widely for collecting insects since the mid-1930s [26] because they are not selective and therefore yield high insect diversities with large amounts of specimens. However, some studies revealed that the malaise trap is selective [27,28]. For example, Aguiar and Santos [27] demonstrated that it collected 2.4 times more males than females, and 20% more species for males than females of Cryptini specimens. They have wide applications and can contribute biological and ecological information to various other fields, including taxonomy and systematics, and biocontrol and biosecurity, when used in a standardized manner [29]. This kind of trap was applied in Thailand to collect flying insects from several areas in the country. It enabled hundreds of new species to be recorded in more than 108 publications and reported in the TIGER project. These included a new black fly species of the rare subgenus *Montisimulium* Rubtsov, and a new record of *S. bishopi* in Thailand, as well as several groups of insects and spiders [12,30,31,32,33,34,35,36,37]. 

Although several ecological studies of the aquatic stages of black flies have been reported in Thailand [23,38,39,40,41], and its neighboring countries [9,42,43], little is known in this country about their community structure and distribution in their adult stage (biting and non-biting black flies). Hence, the aim of this study was to investigate the community structure, biodiversity and spatial (elevation) and temporal (seasonal) distribution of adult black flies in tropical rain forests, by using malaise traps in Doi Inthanon National Park, northern Thailand.

## 2. Materials and Methods

### 2.1. Study Area

**Figure 1:**
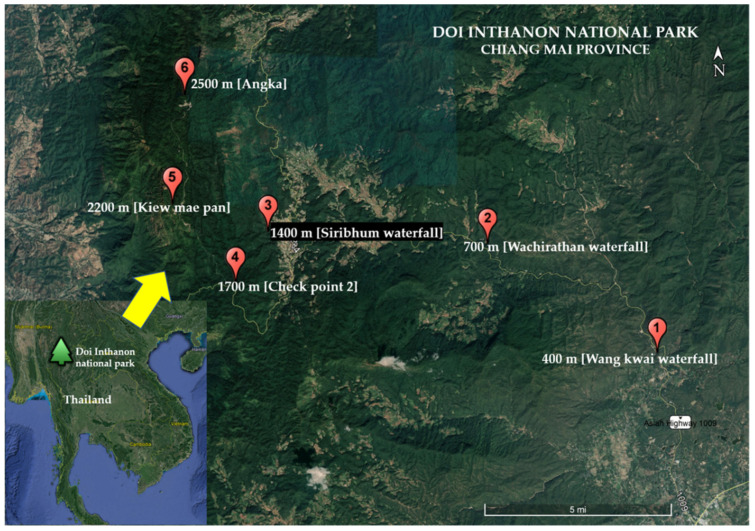
Map of six collection sites along elevational gradients in Doi Inthanon National Park, Chiang Mai province.

Adult black fly specimens were collected monthly using malaise traps (width 100 cm, length 170 cm, height 150 cm) from December 2013 to November 2014. A collection permit (no. 0907.4/20861) for this study was issued by the Department of National Parks, Wildlife and Plant Conservation, Bangkok, Thailand. The study was conducted at six elevation sites, (1) low elevations: 400 m and 700 m; (2) mid elevations: 1400 m and 1700 m; and (3) high elevations: 2200 m and 2500 m above sea level (a.s.l.) in Doi Inthanon National Park, Chiang Mai province, northern Thailand (Figure 1). The physical data of each collection site and classification of forest types [44] are presented in Table 1. Black flies were sorted from other insects and preserved in 80% ethanol before their species was identified in the laboratory of the Entomology Section, Queen Sirikit Botanic Garden (QSBGE), Chiang Mai province, Thailand. The seasonal classification followed the Thai Meteorological Department, i.e., rainy (May to October), cold (November to February) and hot (March to April) seasons [38]. 

### 2.2. Species Identification

Identifications of specimens were morphologically made at species level by using the standard keys [12] and additional keys for black flies in Thailand [10,45]. Exceptionally, specimens in the *S. asakoae* species-group in the subgenus *Gomphostilbia* and those in the *S. striatum* species-group in the subgenus *Simulium* were identified at species-group level, since females of most species of these two species-groups are considerably difficult to separate at species level due to the close similarities of their morphological characteristics [45]. When formally named species are known to consist of more than two cytoforms (or cytospecies), genoforms or morphoforms, they are referred to as species complex [8,11,45]. Representative specimens were deposited at the Entomology Section, QSBGE, Chiang Mai province, Thailand.

### 2.3. Statistical Analyses

The species richness and abundance of black flies collected monthly from each site were recorded. Due to the different number of traps operating among the collection sites, the mean value was used for analysis on spatiotemporal abundance variation and diversity parameters. Relative abundance (percentages) was calculated by dividing the total number of species occurred by the total number of adults. The percentage of species occurrence (% SO) was calculated by determining the number of sites where species were collected and dividing it by the total number of sample sites (*n* = 6) [40]. All data were analyzed by PAST version 4.03 [46]. The diversity parameters of each collecting site included Shannon_H, Simpson_1-D, Dominance_D, Evenness_e^H/S, and Equitability_J. The Chao1 richness estimator was used to estimate the total species richness on Doi Inthanon National Park. The species accumulation curve (individual-based rarefaction) was based on the Shannon_H index, and species richness was used for comparing biodiversity between collection sites and assessing sampling adequacy. Spatiotemporal abundance and richness variation of the black flies collected at each site, including the important six human-biting species, were visualized as a contour map on the elevation/month grid. Multivariate statistic, unweighted pair-group arithmetic averaging (UPGMA) by the Bray–Curtis (two way) similarity index and correspondence analysis (CA) were used to compare and describe the distribution of black fly species associated with collection sites. Correlation analysis was used to test the significant relationship of species richness−elevation. Individual rarefaction was used to compare the diversity between collection sites. Statistical significance was set at *p* < 0.05.

## 3. Results

### 3.1. Community Structure, Species Composition and Biodiversity of Black Flies 

**Figure 2:**
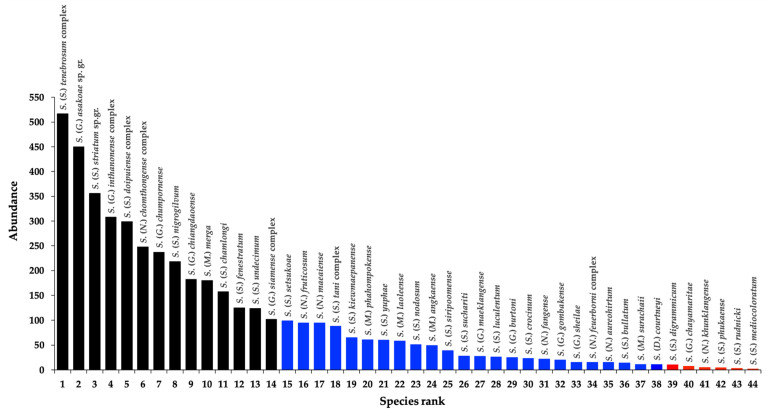
Abundance of black fly species distributed in Doi Inthanon National Park. Black bars represent the species found with more than 100 specimens. Blue bars represent the species found with a range of between 10 and 100 specimens. Red bars represent the species found with ≤ 10 specimens.

In total, 9406 adult black flies belonging to 44 species of five subgenera were trapped in six collection sites: *Daviesellum* Takaoka and Adler (one species, 2%), *Gomphostilbia* Enderlein (10 species, 23%), *Montisimulium* Robtsov (five species, 11%), *Nevermannia* Enderlein (seven species, 16%) and *Simulium* Latreille s. str. (21 species, 48%). Of these, 14 species (32%) were the most abundant, in which more than 100 specimens were found for each one. A range of between 11 and 100 specimens were found for each of 24 species (54%), and six species (14%) were considered as rare, representing ≤ 10 specimens for each one (Figure 2). The most relatively abundant taxon was *S*. *tenebrosum* complex (11.1%), followed by the *S*. *asakoae* species-group (9.6%), the *S*. *striatum* species-group (7.7%), *S*. *inthanonense* complex (6.6%), *S*. *doipuiense* complex (6.4%), *S*. *chomthongense* complex (5.3%), *S*. *chumpornense* (5.1%) and *S*. *nigrogilvum* (4.1%). These eight taxa accounted for 57% of all collected specimens. The most frequent taxa at all of the sites (100% SO) were the two human-biting species—*S*. *nigrogilvum* and the *S*. *asakoae* species-group—followed by *S*. *inthanonense* complex, *S*. *angkaense*, *S*. *chamlongi*, *S*. *fenestratum*, the *S*. *striatum* species-group and *S*. *tani* complex, representing the same percentage of species occurrence (50% SO) (Table S1).

**Figure 3:**
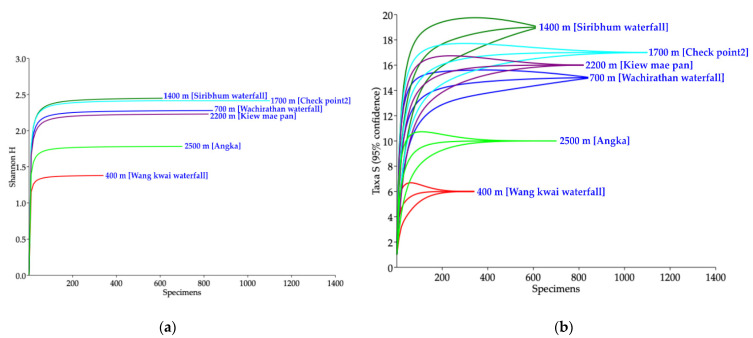
Biodiversity comparison of black flies collected among six collection sites in Doi Inthanon National Park. (**a**) Individual-based rarefaction curves (Shannon_H index), and (**b**) species richness.

Forty-four species were observed, whereas the Chao1 richness estimator suggested 190 species in Doi Inthanon National Park. The biodiversity comparison among collection sites indicated that six of them were well-sampled (Figure 3a,b). Both the Shannon_H index and species richness were highest at an elevation of 1400 m, followed by 1700 m, 2200 m, 700 m, 2500 m and 400 m, with each representing 19, 17, 16, 15, 10 and 6 species, respectively (Table S1, Figure 3a,b).

The trend of richness localities (Table S1), and all diversity parameters (Table S2) of black flies in Doi Inthanon National Park were highest at mid (1400 m and 1700 m) and lowest at low (400 m) elevations. Species richness did not correlate with elevation, and the difference between sites and seasons was insignificant (within each site) (r = 0.273, *p* = 0.600). The evenness_ e^H/S and Equitability_J among the collection sites were a little different, ranging between 0.58 to 0.67 and 0.77 to 0.86, respectively. 

The Bray–Curtis index showed 57%, 48% and 62% similarity of black fly distribution between two sites at low (400 m and 700 m), mid (1400 m and 1700 m) and high (2200 m and 2500 m) elevations, respectively. The ecotone site at low elevation (700 m) shared 53% and 48% similarity with those at mid (1400 m and 1700 m) and high (2200 m) elevations, respectively.

**Figure 4:**
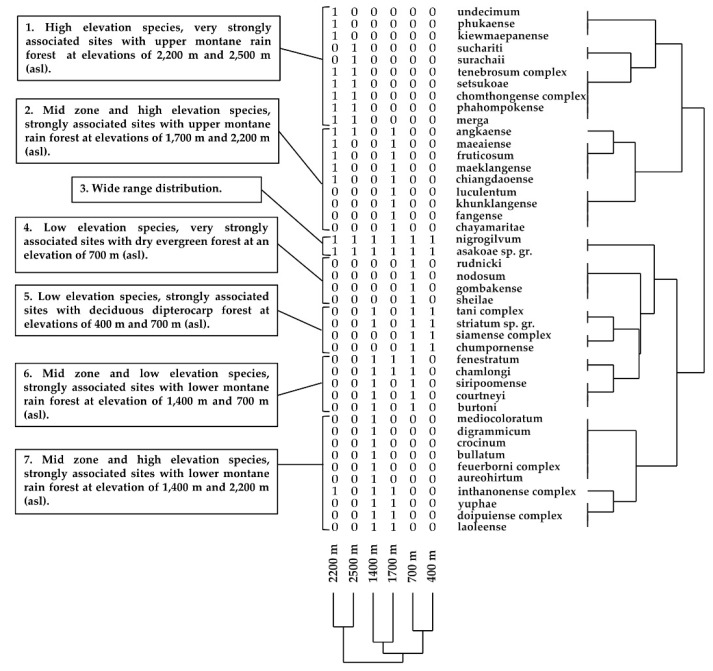
Cluster analysis using the Bray–Curtis resemblance coefficient and UPGMA to produce the dendrogram (Copen. Corr = 0.7728), based on species distribution along six elevational gradients in Doi Inthanon National Park.

**Figure 5:**
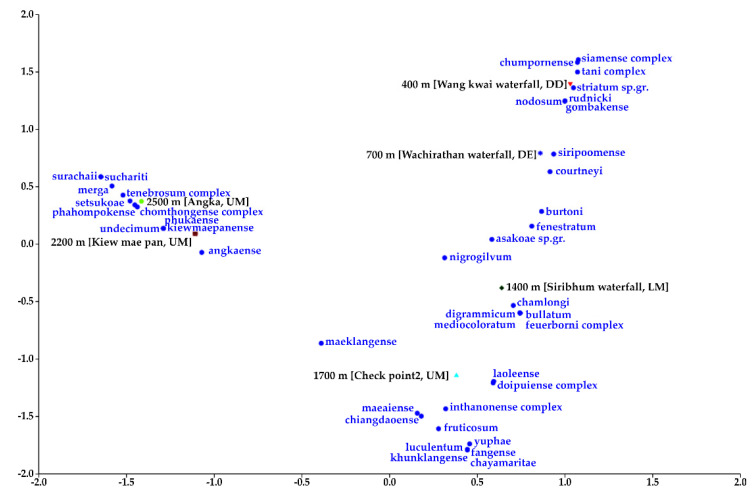
Ordinate diagram of the CA of the 44 black fly species distributed in six collection sites, which correlated to the elevational gradients and forest types of Doi Inthanon National Park. DD: deciduous dipterocarp forest; DE: dry evergreen forest; LM: lower montane rain forest; UM: upper montane rain forest.

Cluster analysis produced seven groups (Figure 4 and Figure 5). Group 1 (10 species) strongly correlated with upper montane rain forest at high elevations (2200 m and 2500 m). Ten species were restricted to both sites with 100% SO. Group 2 (nine species) strongly correlated with upper montane rain forest at mid elevation (1700 m). Some species were found at a high elevation (2200 m). In Group 3, *S*. *nigrogilvum* and the *S*. *asakoae* species-group were common in a wide range of elevations. Group 4 (four species) strongly correlated with dry evergreen forest at low elevation (700 m). Four species were restricted to only this site with 100% SO. Group 5 (four species) strongly correlated with deciduous dipterocarp forest at low elevations (400 m and 700 m). Two of four species were distributed also at mid elevation (1400 m). Group 6 (five species) strongly correlated with lower dry evergreen forest at low (700 m) and mid (1400 m and 1700 m) elevations. Group 7 (10 species) strongly correlated with lower montane rain forest at mid (1400 m to 1700 m) and high (2500 m) elevations.

### 3.2. Spatial (Elevation) and Temporal (Seasonal) Distribution at Each Site 

**Figure 6:**
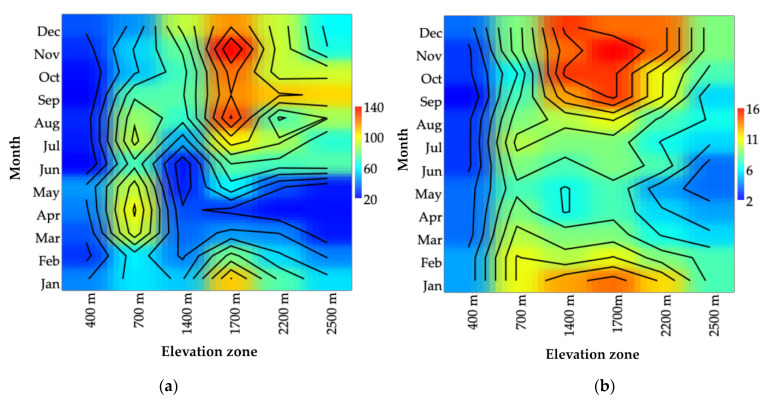
Spatiotemporal variation of black flies in Doi Inthanon National Park. (**a**) Seasonal abundance. (**b**) Seasonal species richness.

A similar trend of adult black fly abundance, collected from each season, was observed. A high seasonal abundance was recorded in the rainy season (May to October) and the lowest in the hot season (March and April) in all of the elevation sites (Table S1, Figure 6a). Seasonal species richness was highest at 1400 m in the cold season (18 species), while an equal number of species was observed at 1700 m and 2500 m in the rainy and cold seasons (Table S1, Figure 6b).

Abundance increased at 400 m elevation from December of the cold season to its highest in May of the early rainy season. It started to increase at 700 m in the hot to late rainy season (March to September), peaked in April, and then decreased to its lowest in December, and started to appear at 1400 m in the mid-rainy to mid-cold season (August to December). The highest number of flies was recorded in December, while the lowest was recorded in June. Abundance started increasing at 1700 m in the mid-rainy season (July). The lowest number of flies was recorded in the hot season (April). Abundance started occurring at 2200 m during the rainy to cold seasons (June to January), whereas the lowest number of flies was recorded in the hot season (April). It started appearing at 2500 m in the rainy season (June to October) and peaked in September (Figure 6).

### 3.3. Distribution of Six Human−Biting Species

**Figure 7:**
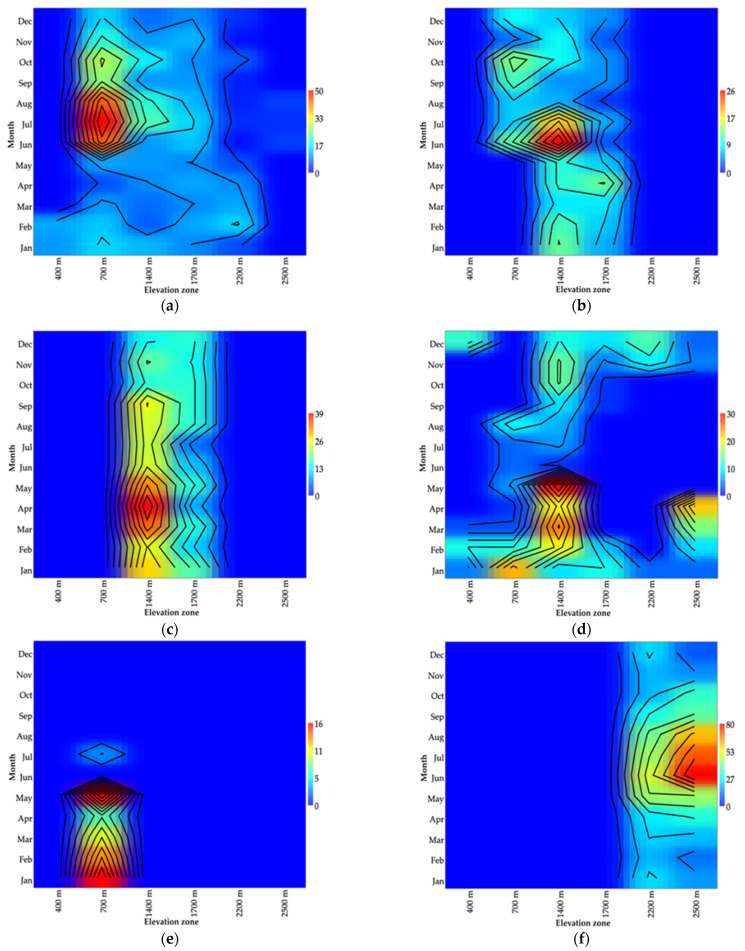
Spatiotemporal variation in abundance of six human-biting species collected along six elevational gradients at Doi Inthanon National Park. (**a**) *Simulium asakoae* species-group. (**b**) *S. chamlongi*. (**c**) *S. doipuiense* complex. (**d**) *S. nigrogilvum*. (**e**) *S. nodosum*. (**f**) *S. tenebrosum* complex.

Species in the *S*. *asakoae* species-group were found in all of the collection sites and predominantly at low elevation (700 m). The high numbers of this species were recorded in the rainy season in almost all of the sites, except for those at 400 m, which were found only in the cold season (Figure 7a). *S. chamlongi* was distributed at low and mid elevations (700 m, 1400 m and 1700 m) and predominantly in the rainy season in all of the sites (Figure 7b). *S*. *doipuiense* complex was found only at mid elevations (1400 m and 1700 m), at which the highest numbers of flies were recorded in the rainy season (Figure 7c). *S*. *nigrogilvum* was collected in all of the six sites and the greatest numbers of flies were recorded at 1400 m in the rainy and cold seasons, followed by 700 m, 1700 m, 2200 m, 2500 m and 400 m elevations, respectively, of which the latter five sites showed the highest numbers of this species in the cold season (Figure 7d). *S*. *nodosum* was found only at low elevation (700 m) in all seasons (Figure 7e). *S. tenebrosum* complex only occurred at high elevations (2200 m to 2500 m) and the highest numbers of flies were recorded in the rainy season (Figure 7f).

## 4. Discussion

### 4.1. Community Structure, Species Composition and Biodiversity of Black Flies

Doi Inthanon National Park is an important biodiversity hotspot for sustainable insect conservation, including black flies [2,22,23,24,40,47,48]. It has a diverse topology with a complex microhabitat and abundance of forest [49], resulting in good-quality flowing streams and the provision of suitable breeding sites for various kinds of insects. In this study, 79% (44/56) of black fly species recorded in Doi Inthanon National Park, which account for 32% (44/139) of total species reported in Thailand, were collected by using malaise traps. In addition, the results of this study showed an estimated 190 black fly species, indicating that, in future, several new species and new records might be discovered in Doi Inthanon National Park. This assumption was supported by the occurrence of several cryptic and species complexes, which have been reported from this area [48,50,51,52,53,54,55,56,57].

### 4.2. Spatial and Temporal Distribution at Each Site

The abundance of black flies in Doi Inthanon National Park reached its highest point during the rainy season, then declined in the cold season and reached its lowest in the hot season. This was because many habitats were not only occupied permanent streams, but also seasonal running streams that supported high numbers of flies in the rainy season. This finding was in agreement with a previous study by Choochote et al. [2], who collected adult black flies using a human-baited method from different elevations (400 m and 2460 m). In contrast, the peak was recorded in Doi Pha Hom Pok during the cold season, as the hand-sampling method was used [39,40]. Similar to previous studies [38,39,40], this study found the peak of seasonal species richness in the cold season, but it was slightly different in a number of species at 1700 m and 2500 m elevations in the rainy season, and low richness was observed in the dry season. Conversely, high species richness was recorded at 700 m elevation during the rainy season, suggesting that the topology of the study site may influence the species richness. The relationship between species richness and elevations is similar to most other regions, for example, “global patterns” that are indicated by a humped curve. A high value of biodiversity occupies the middle elevation zone in many areas, due to the effect of a transition zone or ecotone, which is the optimum condition for many resources that are suitable for several organisms [9,36,37,39,42,43,58,59,60,61,62,63]. While a negative relation was reported from the Andes Mountains in Colombia, the richness was decreased by elevation of between 1800 m and 4750 m, where snow covered the summits [64]. The distribution pattern of the 44 adult black fly species observed along the six elevational gradients in this Park is in agreement with that in previous reports on black fly ecology in Thailand and other countries [9,13,39,40,41,65]. For instance, Srisuka et al. [39] reported that variations in elevation and seasonal conditions had significant effects on the distribution of black fly species collected in Doi Pha Hom Pok. Ya’cob et al. [42] reported the occurrence of four common black fly species related to elevation in the Malaysian Peninsular. Likewise, Hadi et al. [9] found that the distribution of certain black fly species was different according to the elevation of tea plantations in Puncak Bogor, Indonesia. Temperatures have been considered an essential factor in black fly distribution at different elevations [42,65], which affect the characteristics of the aquatic environment [66].

*Simulium* (*D*.) *courtneyi*, one of the two member species of the subgenus *Daviesellum* Takaoka and Adler, was found in this study and its highest abundance was recorded in the rainy season. This study placed malaise traps near Wachirathan and Siribhum waterfalls (Chiang Mai province), where both have fast-flowing water throughout the year, especially during the rainy season. This species was collected from the rocky surface under the waterfalls, and its occurrence was in accordance with a previous report by Takaoka et al. [67]. They found it in the Monthatharn waterfall, which is large and fast-flowing, and located at 730 m elevation in Chiang Mai province.

Ten species members belonging to the subgenus *Gomphostilbia* Enderlein were found from various localities, which accounted for 23% of the total species collected in this study. *S*. *burtoni*, *S*. *gombakense*, *S*. *sheilae* and *S*. *siamense* were found mostly at low elevations (400 m to 700 m), in which their breeding sites were associated with large rivers or the tributaries of mainstreams [1,22,40,42]. Similar to the study by Srisuka et al. [40], *S*. *burtoni* and *S*. *gombakense* were abundant in the cold season. The predominance of *S*. *sheilae* and *S*. *siamense* in the rainy season was in accordance with previous reports in northeastern Thailand [38,40]. *S*. *chumpornense* represented 5.1% of the total flies collected and was also found at low elevations (400 m to 700 m) throughout the year. A high abundance was observed at Wachirathan waterfall (700 m) in the hot season, as reported previously [40,68]. Surprisingly, blooming of this species was observed in the short time period of the hot season in several areas of northern Thailand, especially after the forests had been burnt by fire. It was suspected that carbon dioxide might attract black flies.

Five species members of the subgenus *Montisimulium* Robtsov were found, which represented 11% of the total species collected in this study. Four of them were reported originally from Doi Inthanon National Park and the remaining species, *S*. *phahompokense*, was reported from Doi Pha Hom Pok. These five species were found at mid-high elevations (1400 m to 2500 m) with high abundance in the cold season, which supports previously published reports further [39,40,69,70,71]. The species members of this subgenus in Thailand were found in several localities at high elevation, i.e., Chong Yen (Khamphaeng Phet province), Doi Phuka (Nan province), Phulang Ka waterfall (dry season) and Ban Lek (Phayao province), and Mae klong Yai Village (Tak province) (personal data), the same as reported in other countries [72,73,74,75].

Seven species members of the subgenus *Nevermannia* Enderlein were collected, and they accounted for 16% of the total species collected in this study. *S*. *chomthongense* complex was distributed at high elevation (2200 m to 2500 m), with high abundance in the rainy season. This species strongly correlated with small highland streams and low-temperature water at Doi Pha Hom Pok, as in several species of this subgenus, which distributes in several highland areas of Thailand [39,40].

A total of 21 species members of the subgenus *Simulium* Latreille s. str. were found, which represented 48% of the total species collected in this study. The subgenus *Simulium* is the largest subgenus in Thailand, comprising 54 species. Moreover, some species of this subgenus are medically important flies as biters [2,14,18] and pests of water buffalo, chicken and other domestic animals [2,16,17,76]. The members of this subgenus have a wide distribution range between small lowland and large up to summit streams, and they have a high abundance in various seasons. The most predominant taxa found at low elevation was the *S*. *striatum* species-group, which comprises five species [12], including three new species (*S. wangkwaiense*, *S. tadtonense* and *S. maeklongkeense*) that have been discovered recently [13]. This species group was found in many areas of Thailand [38,39,40], the Oriental region, Taiwan, China, Japan and South Korea [12]. The abundance of this species group varied by elevational gradients from 400 m to 1400 m and had high numbers in the rainy season. Similar to other reports, this species group was associated with large lowland streams with fast-flowing currents [23,39,41]. *S*. *bullatum*, *S*. *crocinum*, *S*. *digrammicum* and *S*. *mediocoloratum* were found only at mid elevation (1400 m). Aquatic stages of *S*. *bullatum* correlated with fast-flowing water and bed rock in small to large waterfalls in many places of Thailand. It is interesting that this species can breed in calcareous waterfalls [39,40]. This study found only aquatic stages of *S*. *crocinum*, *S*. *digrammicum* and *S*. *mediocoloratum* in small canals (10 cm to 30 cm wide) near the trapping area, but not in large streams (10 m to 20 m wide) or waterfalls (30 m wide). These species also were found in grass trailing and the rock surface at Pha Samran waterfall, which was approximately 35 km away from the trapping area. *S*. *kiewmaepanense*, *S*. *phukaense* and *S*. *undecimum* were found only at high elevation (2200 m) in Kiew Mae Pan. A high abundance of these species was observed in the rainy season, as previously reported [77]. *S*. *phukaense* was reported originally from large waterfalls at 1250 m in Nan province [78]. However, the characteristic types of localities were different from this study. It was assumed that this species might fly up from its breeding stream located below this study site in the west (5 km distance). *S*. *suchariti* was found only at the mountain summit (2500 m) in small numbers in the rainy and cold seasons. This was in agreement with a species report by Choochote et al. [2], who collected two specimens with a sweep net in over a year [2]. This species was restricted to only the summit stream of Doi Inthanon National Park, where larvae and pupae were collected.

### 4.3. Distribution of Six Human-Biting Species

The highest relatively abundant species was *S*. *tenebrosum* complex, which represented 11.1% of the total black flies collected. Similar to previous studies, this species was found at high elevations (2200 m to 2500 m) and occurred throughout the year, in which high densities were observed during the rainy season (May to October) [2,23]. Furthermore, it was found at 2100 m in Doi Pha Hom Pok, Chiang Mai province [39]. The two common species—the *S*. *asakoae* species-group and *S*. *nigrogilvum*—have a wide range of distribution and were found in all of the sites. This finding is consistent with previous studies in Thailand [2,40]. The *S*. *asakoae* species-group was collected from 500 m to 2100 m and high numbers of flies were trapped at mid elevations (1400 m to 1500 m) in Doi Pha Hom Pok [39]. It was more abundant during the rainy season at 250 m in Doi Saket district, Chiang Mai province and has been reported as a possible natural vector of filarial worms [17]. In addition, it has been recorded in other Asian countries [8,12]. *S*. *nigrogilvum* is distributed at mid to high elevations (250 m to 2460 m) in northern, western and central Thailand [2,15,16,18,39]. *S*. *chamlongi* was found between 700 m and 1700 m, and large numbers were recorded at the Siribhum waterfall (1400 m), as previously reported [22,39]. *S*. *doipuiense* complex is distributed in a narrow range at 1400 m to 1700 m, in which high numbers were observed at 1700 m. This species was found at 1360 m in Doi Suthep-Pui and also between 800 m and 2460 m at Doi Inthanon National Park [2]. This species also was found at 999 m in northern Vietnam [79]. *S*. *nodosum* was found only at Wachirathan waterfall (700 m) in all seasons, as previously reported in Doi Pha Hom Pok [39]. In addition, this species was found at 860 m and 1360 m in Doi Inthanon National Park [2] and at 250 m in Doi Saket district, Chiang Mai province [17]. It is a human biter and natural vector of *Onchocerca* species in Thailand [16]. Moreover, it has been recorded in India, China, Taiwan, Vietnam and Myanmar [12].

## 5. Conclusions

This study was the first to investigate the community structure, biodiversity and spatiotemporal distribution of adult black flies, by using the malaise trap in northern Thailand. The findings of this study demonstrated the seasonal and temporal variability at the species level (or at species group level). The seasonal abundance and species richness were observed at mid elevations in the rainy and cold seasons. The most predominant species was *S*. *tenebrosum* complex, while the most common taxa were the *S*. *asakoae* species-group and *S*. *nigrogilvum*. Based on the findings of this study, the malaise trap is an effective method, as it is easy to set up at reasonable cost and can capture insects over a long period of time. It is suggested that this trap can be applied to study other insect groups in Thailand.

## Figures and Tables

**Table 1:** Physical data of six collection sites at Doi Inthanon National Park.

Collection Sites	Geographical Coordinates, Elevation (a.s.l. *)	Elevation Zones	No. of Traps	No. of Samplings	Forest Types
Wang kwai waterfall	18°29′57.0″ N 98°40′06.2″ E, 400 m	low	1	12	deciduous dipterocarp forest
Wachirathan waterfall	18°32′27.6″ N 98°36′00.5″ E, 700 m	low	2	24	dry evergreen forest
Siribhum waterfall	18°32′44.4″ N 98°30′53.0″ E, 1400 m	middle	2	24	lower montane rain forest
Check point 2	18°31′39.5″ N 98°29′59.7″ E, 1700 m	middle	2	24	upper montane rain forest
Kiew mae pan	18°33′29.4″ N 98°28′51.7″ E, 2200 m	high	2	24	upper montane rain forest
Angka	18°35′12.8″ N 98°29′14.2″ E, 2500 m	high	3	36	upper montane rain forest
Total			12	144	

* a.s.l. = above sea level.

## Data Availability

The data presented in this study are available in the Supplementary Materials.

## Supplementary Materials

The following are available online at https://www.mdpi.com/article/10.3390/insects12060504/s1: Table S1: species composition, abundance, richness and frequency of fly species occurrence (SO) in streams from black flies collected in Doi Inthanon National Park; Table S2: diversity parameters for black flies at six elevation sites in Doi Inthanon National Park.
