# Supplementary material for: Community Structure, Biodiversity and Spatiotemporal Distribution of the Black Flies (Diptera: Simuliidae) Using Malaise Traps on the Highest Mountain in Thailand
Source: Insects. 2021 May 31;12(6):504. doi: 10.3390/insects12060504 (PMC8229545; doi:10.3390/insects12060504)
Supplement: Supplementary file 1 [file insects-12-00504-s001.zip › Supplementary files/TableS1_Species composition and spatiotemporal in abundant and richness variation.pdf]

Supplementary 1

Table S1: Species composition, abundance, richness and frequency of fly species occurrence (SO) in streams from black flies collected in Doi Inthanon National Park.

Elevations	400 m			700 m			1400 m			1700 m			2200 m			2500 m			Total	RB*	SO** (%)
Seasons	R	C	H	R	C	H	R	C	H	R	C	H	R	C	H	R	C	H			
Species																					
S. (*D*) *courtneyi*	0	0	0	7	2	0	4	0	0	0	0	0	0	0	0	0	0	0	13	0.3	33.3
S. (*G*) *asakoae* sp. gr.	0	7	0	156	32	15	48	23	6	68	45	7	14	15	7	8	0	0	451	9.6	100
S. (*G*) *burtoni*	0	0	0	4	5	3	4	9	0	0	0	0	0	0	0	0	0	0	25	0.5	33.3
S. (*G*) *chayamaritae*	0	0	0	0	0	0	0	0	0	4	3	0	0	0	0	0	0	0	7	0.1	16.6
S. (*G*) *chiangdaoense*	0	0	0	0	0	0	0	0	0	101	41	13	15	13	0	0	0	0	183	3.9	33.3
S. (*G*) *chumpornense*	30	12	43	43	14	96	0	0	0	0	0	0	0	0	0	0	0	0	238	5.1	33.3
S. (*G*) *inthanonense* complex	0	0	0	0	0	0	20	23	2	148	69	18	16	14	0	0	0	0	310	6.6	50
S. (*G*) *sheilae*	0	0	0	11	5	0	0	0	0	0	0	0	0	0	0	0	0	0	16	0.3	16.6
S. (*G*) *siamense* complex	22	12	5	35	23	6	0	0	0	0	0	0	0	0	0	0	0	0	103	2.2	33.3
S. (*G*) *gombakense*	0	0	0	0	19	1	0	0	0	0	0	0	0	0	0	0	0	0	20	0.4	16.6
S. (*G*) *maeklangense*	0	0	0	0	0	0	0	0	0	11	3	0	13	0	0	0	0	0	27	0.6	33.3
S. (*M*) *angkaense*	0	0	0	0	0	0	0	0	0	0	13	0	0	5	0	54	82	13	167	3.6	50
S. (*M*) *laoleense*	0	0	0	0	0	0	2	20	7	7	22	0	0	0	0	0	0	0	58	1.2	33.3
S. (*M*) *merga*	0	0	0	0	0	0	0	0	0	0	0	0	0	32	0	54	82	13	181	3.9	33.3
S. (*M*) *phahompokense*	0	0	0	0	0	0	0	0	0	0	0	0	7	24	2	12	15	0	60	1.3	33.3
S. (*M*) *surachaii*	0	0	0	0	0	0	0	0	0	0	0	0	0	0	0	2	8	0	10	0.2	16.6
S. (*N*) *aureohirtum*	0	0	0	0	0	0	11	4	0	0	0	0	0	0	0	0	0	0	15	0.3	16.6
S. (*N*) *chomthongense* complex	0	0	0	0	0	0	0	0	0	0	0	0	102	32	11	75	26	2	248	5.3	33.3
S. (*N*) *fangense*	0	0	0	0	0	0	0	0	0	8	14	0	0	0	0	0	0	0	22	0.5	16.6
S. (*N*) *feuerborni* complex	0	0	0	0	0	0	3	12	0	0	0	0	0	0	0	0	0	0	15	0.3	16.6
S. (*N*) *fruticosum*	0	0	0	0	0	0	0	0	0	49	33	4	6	0	3	0	0	0	95	2.0	33.3
S. (*N*) *khunklangense*	0	0	0	0	0	0	0	0	0	5	0	0	0	0	0	0	0	0	5	0.1	16.6
S. (*N*) *maeaiense*	0	0	0	0	0	0	0	0	0	38	34	8	0	16	0	0	0	0	96	2.0	33.3
S. (*S*) *bullatum*	0	0	0	0	0	0	9	6	0	0	0	0	0	0	0	0	0	0	15	0.3	16.6
S. (*S.*) *chamlongi*	0	0	0	35	6	0	31	24	6	27	22	7	0	0	0	0	0	0	158	3.4	50
S. (*S.*) *crocinum*	0	0	0	0	0	0	14	9	0	0	0	0	0	0	0	0	0	0	23	0.5	16.6
S. (*S.*) *digrammicum*	0	0	0	0	0	0	7	3	0	0	0	0	0	0	0	0	0	0	10	0.2	16.6
S. (*S.*) *doipuiense* complex	0	0	0	0	0	0	70	51	25	79	65	9	0	0	0	0	0	0	299	6.4	33.3
S. (*S.*) *kiewmaepanense*	0	0	0	0	0	0	0	0	0	0	0	0	32	24	10	0	0	0	66	1.4	16.6
S. (*S.*) *luculentum*	0	0	0	0	0	0	0	0	0	13	14	0	0	0	0	0	0	0	27	0.6	16.6
S. (*S.*) *mediocoloratum*	0	0	0	0	0	0	0	2	0	0	0	0	0	0	0	0	0	0	2	0.0	16.6
S. (*S.*) *nigrogilvum*	0	9	1	20	20	5	32	33	16	5	34	0	0	25	0	0	11	9	220	4.7	100
S. (*S.*) *nodosum*	0	0	0	18	17	16	0	0	0	0	0	0	0	0	0	0	0	0	51	1.1	16.6
S. (*S.*) *phukaense*	0	0	0	0	0	0	0	0	0	0	0	0	2	2	0	0	0	0	4	0.1	16.6
S. (*S.*) *fenestratum*	0	0	0	44	17	13	0	11	6	15	17	4	0	0	0	0	0	0	127	2.7	50
S. (*S.*) *rudnicki*	0	0	0	1	0	2	0	0	0	0	0	0	0	0	0	0	0	0	3	0.1	16.6
S. (*S.*) *setsukoae*	0	0	0	0	0	0	0	0	0	0	0	0	20	9	6	38	18	8	99	2.1	33.3

**Elevations**	**400 m**			**700 m**			**1400 m**			**1700 m**			**2200 m**			**2500 m**			**Total**	**RB***	**SO** (%)**
**Seasons**	**R**	**C**	**H**	**R**	**C**	**H**	**R**	**C**	**H**	**R**	**C**	**H**	**R**	**C**	**H**	**R**	**C**	**H**			
**Species**																					
S. (*S.*) *siripoomense*	0	0	0	22	7	0	0	9	1	0	0	0	0	0	0	0	0	0	39	0.8	33.3
S. (*S.*) *striatum* sp. gr.	90	63	17	52	33	36	47	17	1	0	0	0	0	0	0	0	0	0	356	7.7	50
S. (*S.*) *suchariti*	0	0	0	0	0	0	0	0	0	0	0	0	0	0	0	20	8	0	28	0.6	16.6
S. (*S.*) *tani* complex	14	26	7	12	16	1	9	2	1	0	0	0	0	0	0	0	0	0	88	1.9	50
S. (*S.*) *tenebrosum* complex	0	0	0	0	0	0	0	0	0	0	0	0	162	65	15	235	29	11	517	11.1	33.3
S. (*S.*) *undecimum*	0	0	0	0	0	0	0	0	0	0	0	0	75	37	12	0	0	0	124	2.7	16.6
S. (*S.*) *yuphae*	0	0	0	0	0	0	0	3	0	33	19	5	0	0	0	0	0	0	60	1.3	33.3
**Seasonal abundance**	**156**	**129**	**73**	**460**	**216**	**194**	**311**	**261**	**71**	**611**	**448**	**75**	**464**	**313**	**66**	**498**	**279**	**56**		**100.0**	
**Seasonal richness**	**4**	**6**	**5**	**14**	**13**	**11**	**15**	**18**	**10**	**16**	**16**	**9**	**12**	**14**	**8**	**9**	**9**	**6**			
**Localities richness**	**6**			**15**			**19**			**17**			**16**			**10**					

* RB = Relative abundance; **SO (%) = Species occurrence; R = Rainy; C = Cold; H = Hot
