# Supplementary material for: Community Structure, Biodiversity and Spatiotemporal Distribution of the Black Flies (Diptera: Simuliidae) Using Malaise Traps on the Highest Mountain in Thailand
Source: Insects. 2021 May 31;12(6):504. doi: 10.3390/insects12060504 (PMC8229545; doi:10.3390/insects12060504)
Supplement: Supplementary file 1 [file insects-12-00504-s001.zip › Supplementary files/TableS2_Diversity information of six collection sites.pdf]

Supplementary 2

Table S2: Diversity parameters for black flies at six elevation sites in Doi Inthanon National Park.

Collection sites	Elevation	No. of species	Shannon_H	Simpson_1-D	Dominance_D	Evenness _e^H/S	Equitability_J	Chao-1
Wang kwai waterfall	400 m	6	1.38	0.6879	0.3121	0.6625	0.7702	21
Wachirathan waterfall	700 m	15	2.289	0.8698	0.1302	0.6579	0.8454	120
Siribhume waterfall	1400 m	19	2.467	0.8868	0.1132	0.6204	0.8378	190
Check point 2	1700 m	17	2.431	0.8889	0.1111	0.6691	0.8582	153
Kiew mae pan	2200 m	16	2.24	0.8496	0.1504	0.587	0.8079	136
Angka	2500 m	10	1.794	0.7751	0.2249	0.6016	0.7793	55

Snannon_H: varies from 0 for communities with only a single taxon to high values for communities with many taxa.
Simpson_1-D: measures 'evenness' of the community from 0 to 1.
Dominance_D: ranges from 0 (all taxa are equally present) to 1 (one taxon dominates the community completely).
Evenness e^H/S; proportion of species present in site, ranging from 0 (few species dominate) to 1 (more equal species).
Equitability_J; ranging from 0 to 1 with evenness being complete.
Chao1, an estimate of total species richness.
